# 4′-phosphopantetheine acts as a potential antioxidant to limit atherosclerotic plaque formation by inhibiting ROS generation

**DOI:** 10.3389/fphys.2022.989105

**Published:** 2022-10-21

**Authors:** Taiyu Zhai, Wenbo Ren, Pingping Wang, Xiumei Hu, Jingyu Wang, Lei Zheng

**Affiliations:** ^1^ Department of Laboratory Medicine, Nanfang Hospital, Southern Medical University, Guangzhou, China; ^2^ Department of Clinical Laboratory, The First Hospital of Jilin University, Jilin University, Changchun, China

**Keywords:** 4′-phosphopantetheine, antioxidant, vascular endothelial injury, ROS, coronary heart disease

## Abstract

Coronary heart disease (CHD) is caused by coronary atherosclerosis and has a high morbidity and mortality rate worldwide. There are challenges in both early screening and treatment of CHD. The appearance and development of CHD is a complex metabolic disorder process. Therefore, to search for new biomarkers of CHD, we analyzed the peripheral blood metabolome in patients with CHD. In the study, a plasma metabolite, 4′-Phosphopantetheine (4-PPanSH), which was discovered by HPLC-MS/MS, as peripheral blood 4-PPanSH decreases, the degree of coronary blockage gradually aggravates. In addition, the 4-PPanSH supplement limited atherosclerotic plaque formation and endothelial injury in mice. Further, in vascular endothelial cells, 4-PPanSH effectively inhibited ROS generation and ox-LDL accumulation. In summary, 4-PPanSH was associated with the degree of coronary stenosis, and the 4-PPanSH supplement reduced atherosclerotic plaque generation, which could be associated with 4-PPanSH acting as a potent antioxidant that inhibits ROS generation and alleviates vascular endothelial injury.

## Introduction

Coronary heart disease (CHD) is caused by atherosclerosis (AS) and is a significant cause of morbidity and mortality worldwide (Tian et al., 2019). CHD patients may not have symptoms at the initial stages, therefore, may not seek medical attention, leading to a gradual aggravation of vascular stenosis and occlusion. However, rupturing of unstable atherosclerotic plaque and severe coronary occlusion can cause serious consequences, including myocardial infarction and sudden cardiac death. These consequences pose a great threat to patients’ life and safety (Zhu et al., 2018). Hence, improving the early diagnosis of CHD is critical for early treatment.

Currently, imaging techniques, such as coronary computed tomography angiography (CCTA) and coronary angiography (CAG), are used primarily to diagnose CHD. However, these techniques are not commonly used as routine screening programs due to their invasiveness, cost, and exposure to ionizing radiation. Therefore, people with high-risk CHD and patients with silent CHD that contribute more than 75% of ischemic episodes cannot be detected in time (Shah et al., 2015; Faroux et al., 2019). In addition, some hematological biomarkers, such as troponin, can be used to guide the diagnosis of CHD in laboratories. Troponins are considered markers of choice for diagnosing myocardial infarction and B-type natriuretic peptide (BNP) with heart failure. However, the specificity and sensitivity of these biomarkers are currently limited, and abnormalities are detected only after the onset of relevant symptoms. These biomarkers are unable to provide an effective risk indication to patients at an early stage; therefore, it is necessary to look for new serological biomarkers for the early diagnosis of CHD.

The occurrence and development of AS or CHD is a complex metabolic disorder process that involves multiple risk factors. The changes in metabolites in peripheral blood play a vital role in the progression of AS (Poznyak et al., 2020). Various metabolites are involved in the progression of AS, including trimethylamine oxide (TMAO) and short-chain fatty acids, which could serve as potential therapeutic targets for AS and diagnostic markers for AS patients (Verhaar et al., 2020). However, most investigations are still at a preliminary stage as these metabolites do not have a clear causal relationship with atherosclerotic cardiovascular disease (ASCVD) (Jia et al., 2019). The Gensini score is a widely used clinical measure to evaluate the extent of coronary artery disease, and this score significantly assesses the condition of CHD patients (He et al., 2021). In this study, metabolome differences were detected after grading them by Gensini score in peripheral blood from patients, thus searching for biomarkers associated with the degree of coronary stenosis and further evaluating whether it is associated with the progression of AS.

## Materials and methods

### Chemical reagents and materials

Fisher Scientific (Massachusetts, United States) supplied acetonitrile (ACN) and methanol (MeOH). Formic acid (FA) was purchased from Merck (Darmstadt, Germany). A Milli-Q water purification system was used to prepare ultra-high-purity water. The standard for 4′-Phosphopantetheine (4-PPanSH) was synthesized and verified by Xinyao Biological Technology (Shenzhen, China). As an internal standard, Reserpine, Carbamazepine-d10 (Car-d10) was purchased from Sigma-Aldrich (Darmstadt, Germany). Yiyuan Biotechnologies (Guangdong, China) provided Dil-ox-LDL. The reactive oxygen species assay kit was purchased from Biosharp Life Science (Anhui, China). Paraformaldehyde (4%) and isopropanol (60%) were provided by Sigma-Aldrich (Darmstadt, Germany), and the modified oil red O staining kit was purchased from Biotime (Shanghai, China).

### Plasma sample collection and extraction

The research was carried out according to the Code of Ethics of the World Medical Association (Declaration of Helsinki), that informed consent was obtained, and the institutional review board approved the study. The Human Research Ethics Committee of the First Hospital, Jilin University, approved the study. In 2021, plasma samples were collected from the First Hospital of Jilin University. All plasma samples were collected before CAG testing, and untargeted metabolome testing was performed on 118 patient samples that underwent CAG testing to determine the extent of coronary stenosis with an initial diagnosis of CHD. Furthermore, 151 samples were collected and used for the quantitative analysis of 4-PPanSH. Before the study, all subjects gave their written informed consent. All plasma samples were obtained after centrifugation of blood in K2 EDTA vacutainer tubes and immediately frozen (-80°C) until sample preparation.

Plasma samples (50 µl), Reserpineor or Car-d10 (IS) working solution (50 µl), and acetonitrile (200 µL) were added to a 1.5 ml Falcon tube, mixed well for 30 s, and centrifuged at 4°C at 15,000 rpm for 10 min. For analysis, the supernatant was injected into the LC-MS/MS.

### Untargeted metabolomics

Metabolite profiling was performed for MRM analysis using a Shimadzu Exion LCTM Ultra-High Performance Liquid Chromatography (UHPLC) system (Kyoto, Japan) with a binary pump, autosampler, thermostatically controlled column compartment, and an auto sample manager coupled with a triple-quadrupole/time-of-flight 5,600 mass spectrometer (TOF-MS) (AB Sciex, Ont, Canada). Metabolites were eluted on a BEH C18 column (100 × 2.1 mm, 1.7 µm; Waters, Ireland). The mobile phase consisted of 0.1% formic acid in water (solvent A) and acetonitrile (solvent B). MS analysis with an electrospray ionization (ESI) source operated in the positive ion mode over a mass range of 50–1,500 Da in the TOF scan mode. The TOF scan time was maintained at 14 min. Analyst TF 1.7.1 software was used to create the file.

### Analysis of 4-PPanSH

4-PPanSH was performed on an LC-MS platform composed of an LC-20AD series HPLC (Shimadzu Corporation, Kyoto, Japan), connected with a QTrap 5,500 mass spectrometer (AB Sciex, Ontario, Canada) and TurboIonSpray™ source. The HPLC system is equipped with a binary pump, a degasser, a column department, and an auto sample manager. A Shim-pack GIST-HP C18 column (150 × 2.1 mm, 3 µm) was used to achieve the separations of 4-PPanSH at 40°C. The mobile phase consisted of 0.1% formic acid in water (solvent A) and acetonitrile (solvent B). The separation gradient program followed: 0.0–1.0 min, (5.0% B); 1.0–4.0 min, (5.0%–95.0% B); 4.0–6.0 min, (95.0% B); 6.0–6.1 min: (95.0%–5.0% B); 6.1–9.0 min (5.0% B). The samples (10 µl) were produced at a 0.4 ml/min flow rate.

The mass spectrometer was equipped with an electrospray ionization (ESI) source that worked in positive ion mode, with the following MS parameters: ion spray voltages: 4500 V; the source temperature: 450°C; and ion source gas: 1 40 psi; ion source gas: 2 40 psi; decluttering potentials: 70 V and collision energies: 4-PPanSH (20 eV), Car-d10 (30 eV). Transitions (m/z) for MRM were 4-PPanSH 359.1→216.2; Car-d10 247.1→204.2. Chromatographic peaks were processed by extracting the ion chromatogram of the analyses at theoretical m/z. Applied Biosystems Analyst software version 1.5 was used to perform data acquisition and integration.

### Analysis of coenzyme A

Human carotid atherosclerotic plaques were derived from patients with ischemic stroke obtained by carotid endarterectomy. A method for detecting coenzyme A has previously been reported (Shurubor et al., 2017). A 5% perchloric acid (200 µl) aqueous solution containing 50 µM DTT was configured, and then frozen human tissue samples of carotid atherosclerotic plaques were added. It was mixed well, vortexed, and homogenized by quickly sonicating for 12 s at 20% amplitude and then centrifuged at 14,000 g for 10 min at 4°C. The plasma sample (50 µl) was added separately to a pre-chilled solution containing 250 µl of 5% perchloric acid and 50 µM DTT. As described, the acid-treated extracts were vortexed and centrifuged. The corresponding calibration curve was used to calculate the CoA concentrations in plasma samples.

### Construction of a mice atherosclerosis model

Charles River (Beijing, China) provided the 12 five-week-old specific pathogen-free (SPF) grade apolipoprotein E deficient (ApoE^−/−^) mice weighing 16–21 g. The mice were divided into two groups. In the first group, mice were fed only a high-fat diet (Xietong Biotechnologies, Jiangsu, China), and in the second group, mice were fed a high-fat diet with an additional 4-PPanSH (10 µg), injected through the tail vein once a week. All mice were fed for a total of 15 weeks. All animal experiments were conducted following the regulations of the Animal Ethics Committee of Jilin University.

### Blood biochemistry analysis

After 15 weeks, the blood of the mice was collected by cardiac puncture to conduct biochemical studies. Blood was centrifuged at 3,000 rpm for 10 min at 4°C to separate serum, which was then stored at −80°C. The Beckman Coulter biochemical auto analyzer (California, United States) was used to measure serum levels of total cholesterol (TC), triglycerides (TG), low-density lipoprotein cholesterol (LDL-C), high-density lipoprotein cholesterol (HDL-C), and blood glucose (Glu).

### Oil-red O staining of frozen sections of the abdominal aorta and aortic arch

Mice aortas were removed, longitudinally cut from the intercostal ostia to the iliac bifurcation, and secured with needles. After that, each aortic specimen was fixed with 4% paraformaldehyde and rinsed with 60% isopropanol. A 60% Oil Red O solution in isopropanol was used to stain the aortic specimens, which were then cleaned with 60% isopropanol. The stained aortic tissues were finally washed with deionized water prior to imaging. The aortic arch tissue was first sectioned at −20°C with a section thickness of 4–6 µm. The sections were soaked and washed with 60% isopropyl alcohol for 10 min and then treated with Oil Red O staining solution for 10 min. After staining, sections were treated with 60% isopropanol for 10 s, rinsed in deionized water, and then counterstained with hematoxylin staining solution for 1 min.

### Reactive oxygen species assay

The manufacturer’s recommendations for ROS detection were followed. Briefly, human umbilical vein vascular endothelial cells were divided into two groups. The first group was induced by adding ox-LDL (100 μg/ml) to produce ROS, and in the second group, 1 μg/ml of 4-PPanSH was added and then incubated at 37°C for 24 h. The H2DCFDA probe (1:1,000) was then added and incubated for 30 min at 37°C. A fluorescent microscope was used to take the photographs, and the fluorescence intensity was detected using a fluorescence microplate reader (Yan et al., 2018).

To detect ROS in mice vascular tissues, the tissues should be washed twice with pre-cooling PBS buffer following the manufacturer’s instructions (Bestbio, China). Plaque tissues or surrounding normal vascular tissues were homogenized twice by adding 1 ml of tissue homogenization buffer using an electric homogenizer. After thorough homogenization, the suspension was centrifuged at 140000 × g for 10 min at 4°C. The protein concentration in the supernatant was detected by BCA, and the fluorescence intensity of the supernatant was detected using a fluorescence microplate reader at 490 nm excitation and 525 nm absorption wavelengths. The ROS content in tissues was expressed as fluorescence intensity/protein concentration.

### Ox-LDL assay

For ox-LDL detection in cells, we used DIL-labeled human ox-LDL (100 μg/ml) co-cultured with HUVECs. After 24 h, the cells were rinsed three times with PBS, and the culture solution was changed with a fresh one. The fluorescence intensity of the samples was detected using a fluorescence microplate reader at 488 nm as excitation wavelength and 530 nm as absorption wavelength.

To detect ox-LDL in tissues, the samples were treated similarly to the detection of ROS, following the manufacturer’s instructions (Finetest, China). Of a diluted sample, 100 µl was added to the test sample wells. The plate was covered and incubated at 37°C for 90 min. Then, 100 µl of biotin-labeled antibody in working solution was added to the wells (standard, test sample, and blank wells). The solution was added at the bottom of each well without touching the sidewall. The plate was covered and incubated at 37°C for 60 min. The lid was removed, and the plate was washed thrice with washing buffer. Of SABC working solution, 100 µl was added to each well, the plate was covered, and incubated at 37°C for 30 min. After washing the plate 5 times with the washing buffer, 90 µl TMB substrate was added to each well, and the plate was covered and incubated at 37°C in the dark for 10–20 min. Of the stop solution, 50 µl was added to each well. The absorbance immediately after adding the stop solution.

### Surface analysis of mice arteries

The mice artery surface morphology was analyzed using a previously reported methodology (Kluza et al., 2021). Briefly, the vascular tissues of mice were dehydrated in gradient ethanol solutions of different concentrations and soaked in hexamethyldisilazane (Sigma Aldrich) for 30 min and air dried. The scanning electron microscope was used to capture images at 3 kV at different magnifications.

### Statistical analyses

The standard error of the mean (SEM) of each group was used to produce the results (*p*-value < 0.05 was considered statistically significant). Perform PLS-DA analysis, heat map, and enriched analysis using metaboanalyst 5.0. Using GraphPad Prism 9.0 software, receiver operating characteristics (ROC) curves were constructed to assess sensitivity, specificity, and respective areas under the curves (AUCs) with 95% CI. To determine the optimal diagnostic cutoff thresholds, the Youden index was used. We determined the optimal cutoff value for diagnosis by maximizing the sum of sensitivity and specificity and minimizing the overall error (SQRT [1–sensitivity] (Zhu et al., 2018) + [1–specificity] (Zhu et al., 2018)) and by minimizing the distance of the cutoff value to the upper left corner of the ROC curve (Wians, 2009).

## Results

### Metabolites that are associated with the differential extent of CHD were screened by untargeted metabolomic

The discovery cohort included a total of 118 participants (Supplementary Table S1), all of whom were admitted with an initial diagnosis of CHD and had undergone coronary angiography. Based on angiographic data and Gensini scores, these participants were further divided into the following five groups (Supplementary Table S2): Two groups of non-CHD patients include normal coronary artery (NON-CA) and coronary arteries having plaque but not forming stenosis group (CA). Three other groups of patients with confirmed CHD include the mild stenosis group (Mild), the moderate stenosis group (Moderate), and the severe stenosis group (Severe). In addition to this, considering that 75% of patients with asymptomatic CHD are not effectively diagnosed, we adopted the NON-CA group as a control.

**FIGURE 1:**
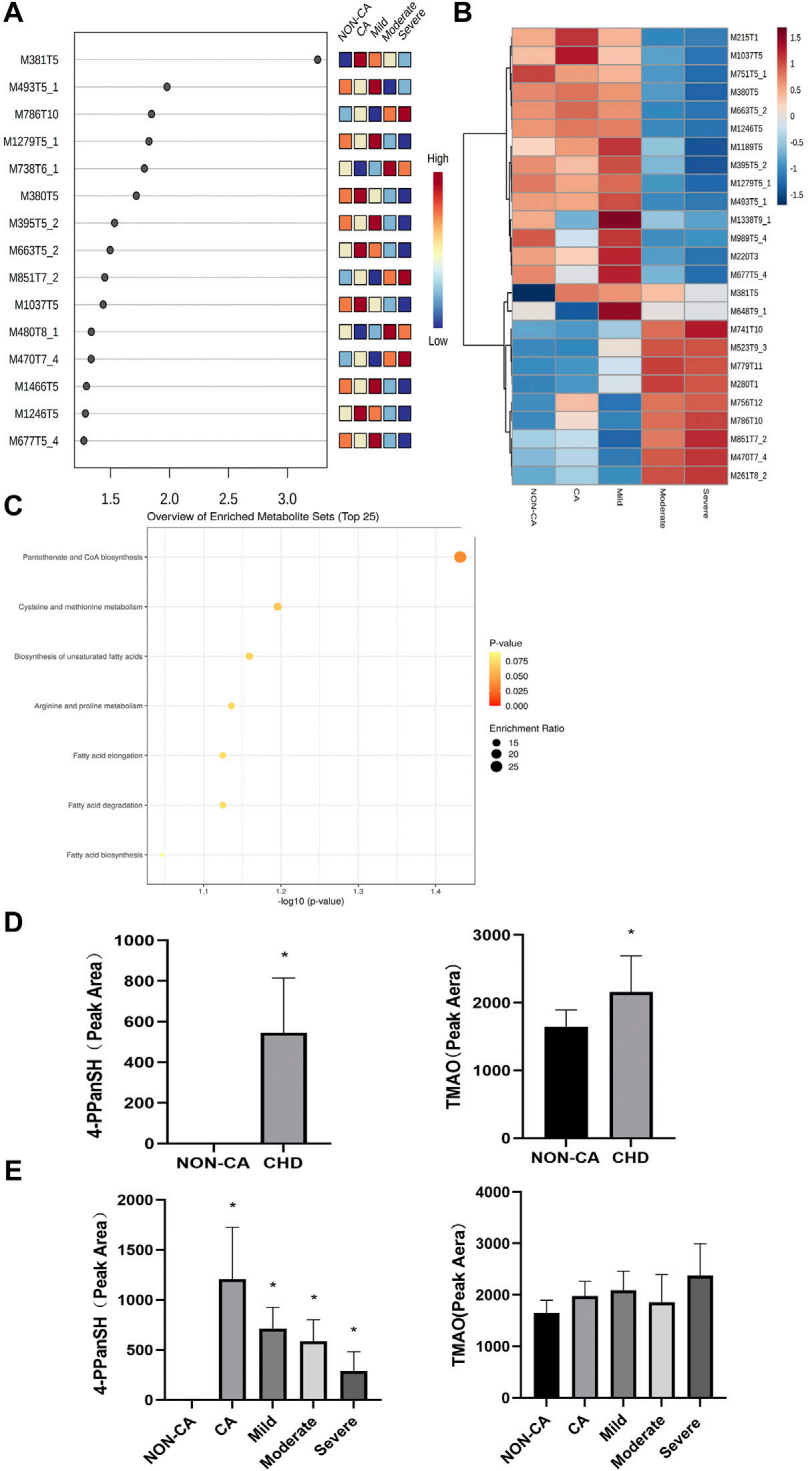
4-PPanSH was associated with various degrees of CHD. **(A)** The VIP score analysis showed that M381T5 was the potential diagnostic marker in five groups. **(B)** Cluster analysis in the discovery cohort. **(C)** Enrichment analysis for differential metabolites revealed that coenzyme A biosynthesis is the most relevant signaling pathway. **(D)** The results of the semiquantitative analysis showed that both 4-PPanSH and TMAO were apparent differences in the NON-CA and CHD groups. **(E)** Semiquantitative analysis showed that 4-PPanSH was associated with different severity of CHD. *Indicates *p* < 0.05 compared to the NON-CA group.

A total of 69 differential metabolites were found through untargeted metabolomics detection by UHPLC-Triple-TOF-MS and database matching. For the PLS-DA analysis, the *p*-value was set at less than 0.05, and the importance of the variable in the projection score (VIP) was set at greater than 1.0 as the screening criterion. The result showed that M381T5 was the differential metabolites in five groups (Figure 1A). The M381T5 metabolite efficiently distinguished samples from five different groups in cluster analysis (Figure 1B). Database matching determined M381T5 as it corresponds to 4′-phosphopantetheine (4-PPanSH). Enrichment analysis revealed that the differential metabolic pathways were most abundant in the coenzyme A biosynthesis signaling pathway (Figure 1C).

We examined the peak area of 4-PPanSH using untargeted metabolomics data to clarify differences in 4-PPanSH expression at different severity levels in CHD patients. To compare the evaluation capacity of 4-PPanSH in CHD, we selected TMAO, which has recently been studied more frequently. The result showed that the content of 4-PPanSH and TMAO was higher in CHD patients than in the NON-CA group (Figure 1D). However, 4-PPanSH expression was associated with the severity of CHD, whereas TMAO did not differ significantly between the groups (Figure 1E). The result indicates that 4-PPanSH has a better evaluation ability for CHD patients with different degrees of the lesion.

4-PPanSH provided better suggestions for potential CHD patients and high-risk groups.

**FIGURE 2:**
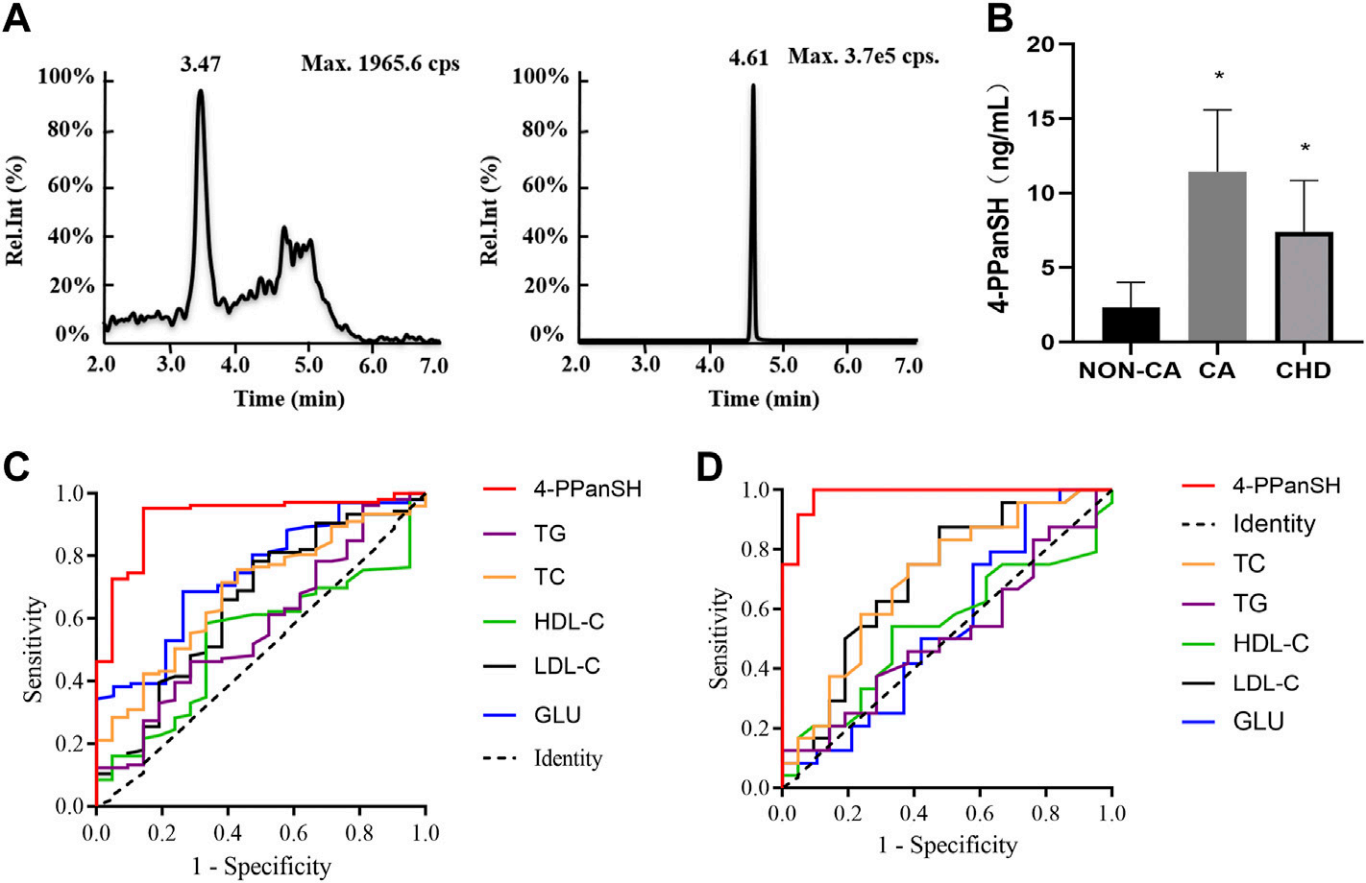
4-PPanSH provided better suggestions for the potential CHD high-risk and CHD groups. **(A)** Representative chromatograms of 4-PPanSH and Car-d10 in human plasma. **(B)** 4-PPanSH was significantly higher in the CA group and decreased after the diagnosis of CHD was confirmed. **(C)** 4-PPanSH was more effective than traditional indices of CHD patients. **(D)** 4-PPanSH was more effective than the traditional indexes of the potential CHD high-risk groups. *Indicates *p* < 0.05 compared to the NON-CA group.

Precise quantification of 4-PPanSH is required to confirm the accuracy as untargeted metabolomics is only a semiquantitative analysis. For further precise quantification of 4-PPanSH, we synthesized 4-PPanSH as standard (Supplementary Figure S1A) and Car-d10 as internal standard (Supplementary Figure S1B); we also developed an analytical method for the quantification of 4-PPanSH using HPLC-Q-Trap-MS (Supplementary Table S3). The HPLC condition can achieve complete chromatographic separation of 4-PPanSH from matrix components, with elution of 2 min–7 min and a total run time of 9 min, allowing high sample throughput (Figure 2A).

The HPLC-MS/MS method was then used to measure 4-PPanSH in the validation cohort (*n* = 151) (Supplementary Table S4). The assay demonstrated that 4-PPanSH levels in peripheral blood samples that were low in normal coronary arteries (NON-CA group) were significantly increased after the appearance of coronary plaque (group CA) but decreased in patients with established CHD (Figure 2B).

Table 1 shows the optimal cutoff value, positive predictive value (PPV), and negative predictive value (NPV) of 4-PPanSH. These results showed that 4-PPanSH could be a potential marker for the diagnosis of both CA and CHD groups with a Youden index of 0.81 and 0.905 and optimal cutoff values of 8.915 and 2.325, respectively (Table 1). Furthermore, we discovered that the area under the curve (AUC) of 4-PPanSH for CHD was 0.92, which was significant compared to other traditional predictors of risk of AS, including TC and LDL-C (Figure 2C). With an AUC of 0.98, 4-PPanSH provided better suggestions for potential CHD high-risk groups (Figure 2D).

**TABLE 1:** Evaluation of the diagnostic efficacy of 4-PPanSH.

Characteristics	CA	CHD
Optimal cut-off value (ng/mL)	2.325	8.915
Youden index	0.905	0.810
PPV (%)	92.300	94.020
NPV (%)	100.000	88.520
Sensitivity (%)	100.000	95.280
Specificity (%)	90.480	85.710
AUC	0.980	0.920
95% CI	0.956–1.000	0.861–0.984

CHD(*n* = 106): coronary heart disease group; CA(*n* = 24): coronary arteries that have plaque but do not form stenosis group; PPV, positive predictive value; NPV, negative predictive value; AUC, area under the curve; 95% CI: 95% confidence intervals.

### 4-PPanSH was related to CHD patients with different degrees of coronary occlusion

**FIGURE 3:**
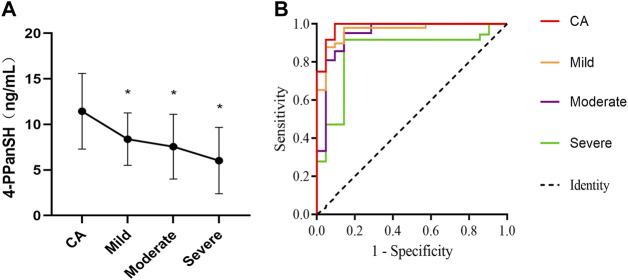
4-PPanSH was related to CHD patients with different degrees of coronary occlusion. **(A)** 4-PPanSH content gradually decreased with increased coronary occlusion. **(B)** 4-PPanSH was associated with the degree of coronary stenosis. *Indicates *p* < 0.05 compared to the CA group.

The 4-PPanSH levels were investigated in peripheral blood from patients with various levels of CHD. The 4-PPanSH levels in the peripheral blood gradually decreased as the degree of coronary occlusion deteriorated (Figure 3A). We also analyzed the cutoff value of 4-PPanSH in peripheral blood from CHD patients of different degrees. These findings suggested that the 4-PPanSH content was related to CHD patients with different degrees of coronary occlusion (Figure 3B, Table 2).

**TABLE 2:** Diagnostic ability of 4-PPanSH in patients with different grades of CHD.

Characteristics	Mild	Moderate	Severe
Optimal cut-off value (ng/mL)	9.280	6.615	4.270
Youden index	0.837	0.810	0.774
PPV (%)	94.120	91.330	91.660
NPV (%)	93.740	91.930	85.710
Sensitivity (%)	97.960	95.240	91.670
Specificity (%)	85.710	85.710	85.710
AUC	0.960	0.950	0.850
95% CI	0.922–1.000	0.876–1.000	0.742–0.964

Mild (*n* = 49): mild stenosis group; moderate (*n* = 21): moderate stenosis group; severe (*n* = 36): severe stenosis group; PPVpositive predictive value; NPV, negative predictive value; AUC, area under the curve; 95% CI: 95% confidence intervals.

### 4-PPanSH could attenuate the formation of AS plaques in mice

**FIGURE 4:**
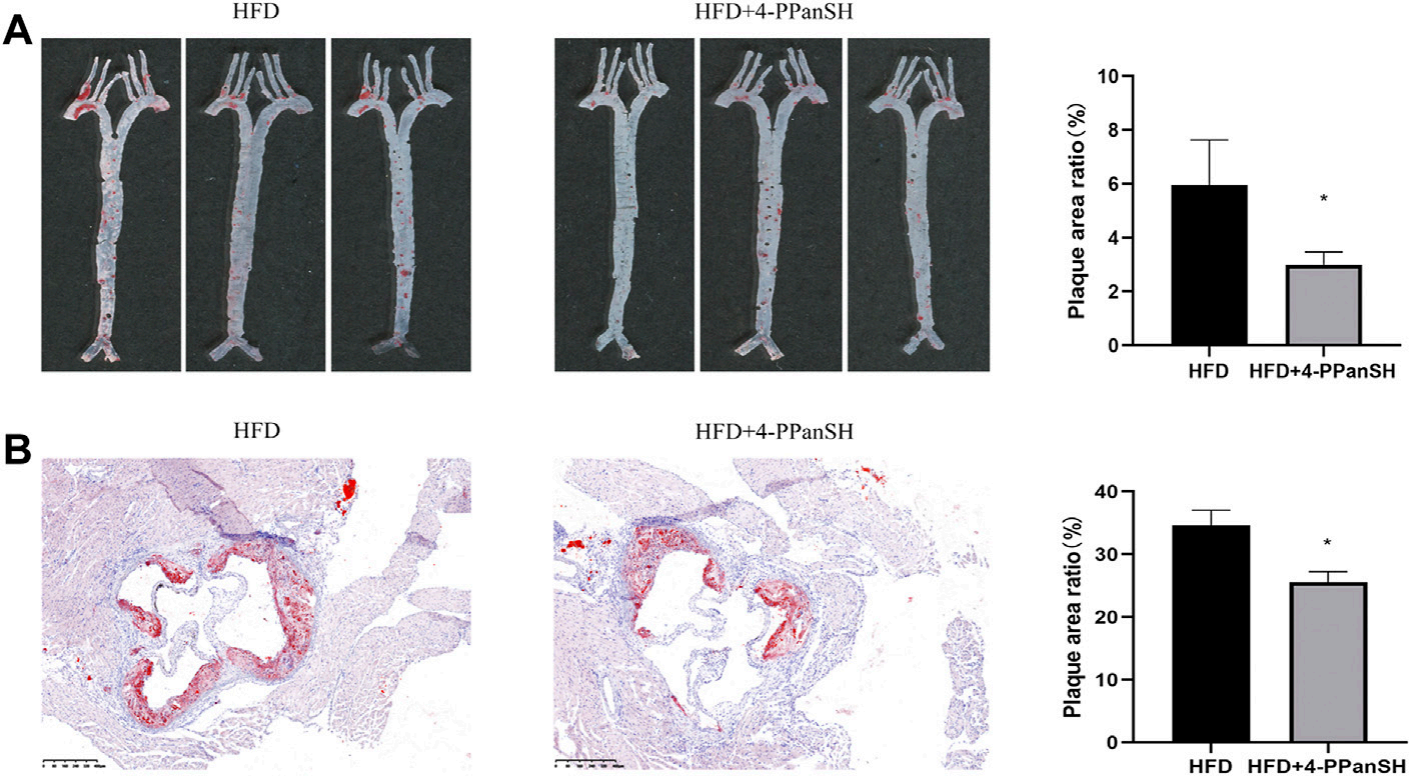
4-PPanSH could attenuate the formation of AS plaques. **(A)** The abdominal aortas of mice were stained with oil red O (*n* = 3). 4-PPanSH could significantly inhibit AS plaque formation in mice. **(B)** 4-PPanSH could alleviate aortic root lesions in mice (*n* = 3). *Indicates *p* < 0.05 compared to the HFD group.

In the above results, the inverse correlation between the 4-PPanSH level and the degree of coronary occlusion was detected, suggesting that 4-PPanSH may have a potential effect on atherosclerotic plaque formation. Therefore, we constructed an AS model using ApoE^−/−^/mice to investigate whether 4-PPanSH affected atherosclerotic plaque formation under high-fat diet conditions (HFD). The result showed that 4-PPanSH significantly reduced the plaque area ratio and decreased root lesions in mice (Figures 4A,B).

### 4-PPanSH could attenuate vascular endothelial injury in mice

**FIGURE 5:**
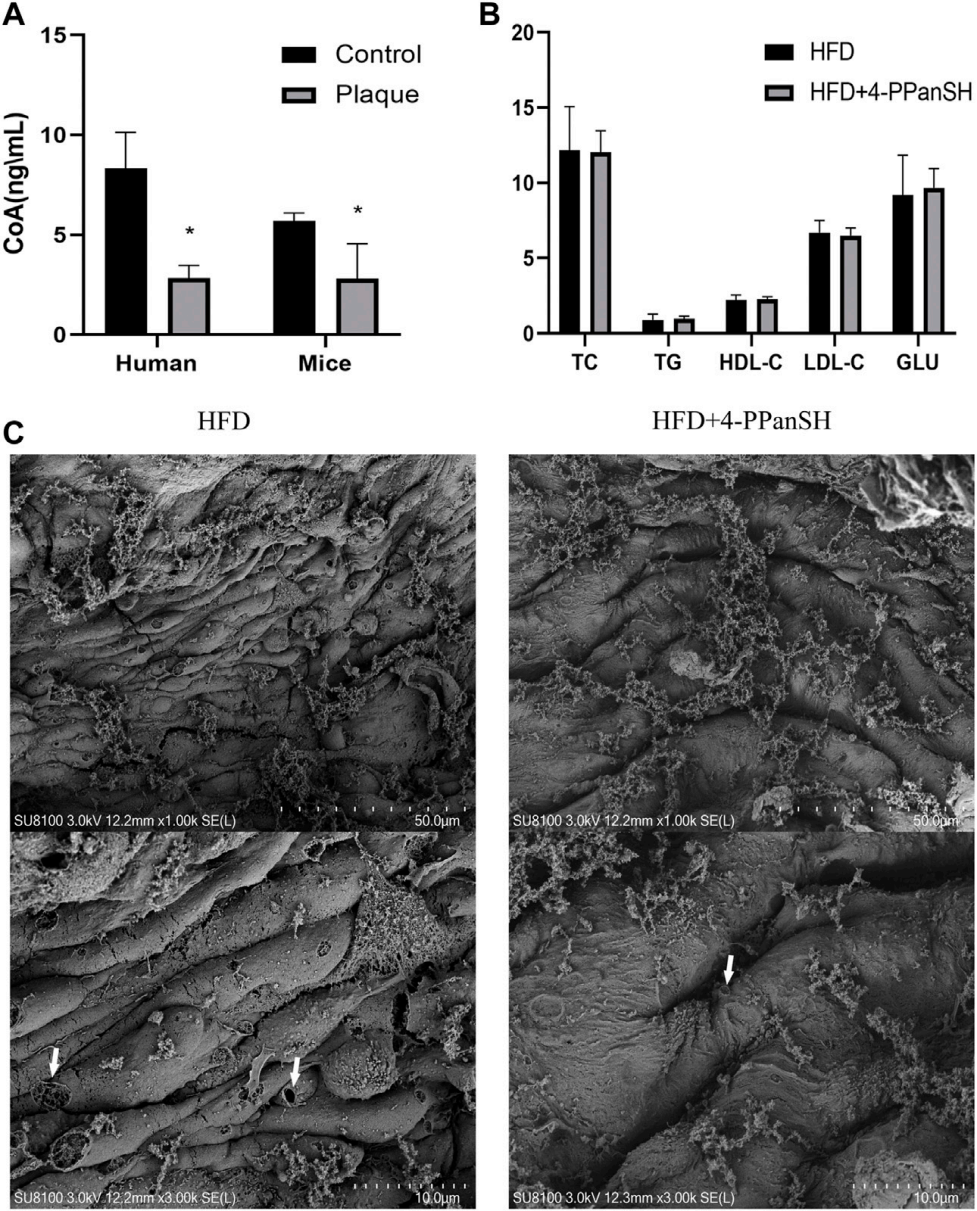
4-PPanSH could attenuate vascular endothelial injury. **(A)** The CoA content in plaques of human rigidified arteries and the abdominal aortas of mice were detected by HPLC-MS and the surrounding normal vascular tissues were used as controls (*n* = 3). CoA in atherosclerotic plaque samples was reduced. **(B)** 4-PPanSH had no apparent effects on blood lipids in mice (*n* = 3). **(C)** 4-PPanSH attenuated the hollow-like injury of the vascular endothelium in mice. *Indicates *p* < 0.05 compared to the control group.

As previously stated, the results of the untargeted metabolome assay showed that the differential metabolites were mostly enriched in the CoA biosynthetic signaling pathway (Table 1). CoA plays a critical role in regulating metabolic activities *in vivo*, while 4-PPanSH is a critical intermediate in CoA synthesis and metabolism. Hence, the effect of the anti-AS plaque generation of 4-PPanSH could possibly be related to CoA. As a result, we first identified the CoA level in peripheral blood without several assay conditions, including the HPLC-MS/MS that we developed. However, the CoA content in human or mice atherosclerotic plaque tissue was significantly lower than surrounding normal vascular tissue (Figure 5A). Considering the crucial role of CoA in the lipid metabolism process, we measured conventional lipid indicators, such as TC and TG, in the peripheral blood of AS model mice. The result showed that 4-PPanSH had no significant effect on blood lipids (Figure 5B), and the effect of the anti-AS plaque generation of 4-PPanSH was independent of improving dyslipidemia.

Along with lipid metabolism disorders, a vascular endothelial injury is a key event in the pathogenesis of AS. In this study, 4-PPanSH significantly reduced hollow-like vascular endothelial injury in mice while testing the effect of 4-PPanSH on vascular endothelial injury in mice. Endothelial cells in the HFD group were elongated with bulging cell membranes and many perforations, yet 4-PPanSH could effectively reduce this lesion (Figure 5C). Hence, the anti-AS effect of 4-PPanSH was associated with alleviation of vascular endothelial injury.

### 4-PPanSH could inhibit ROS generation

**FIGURE 6:**
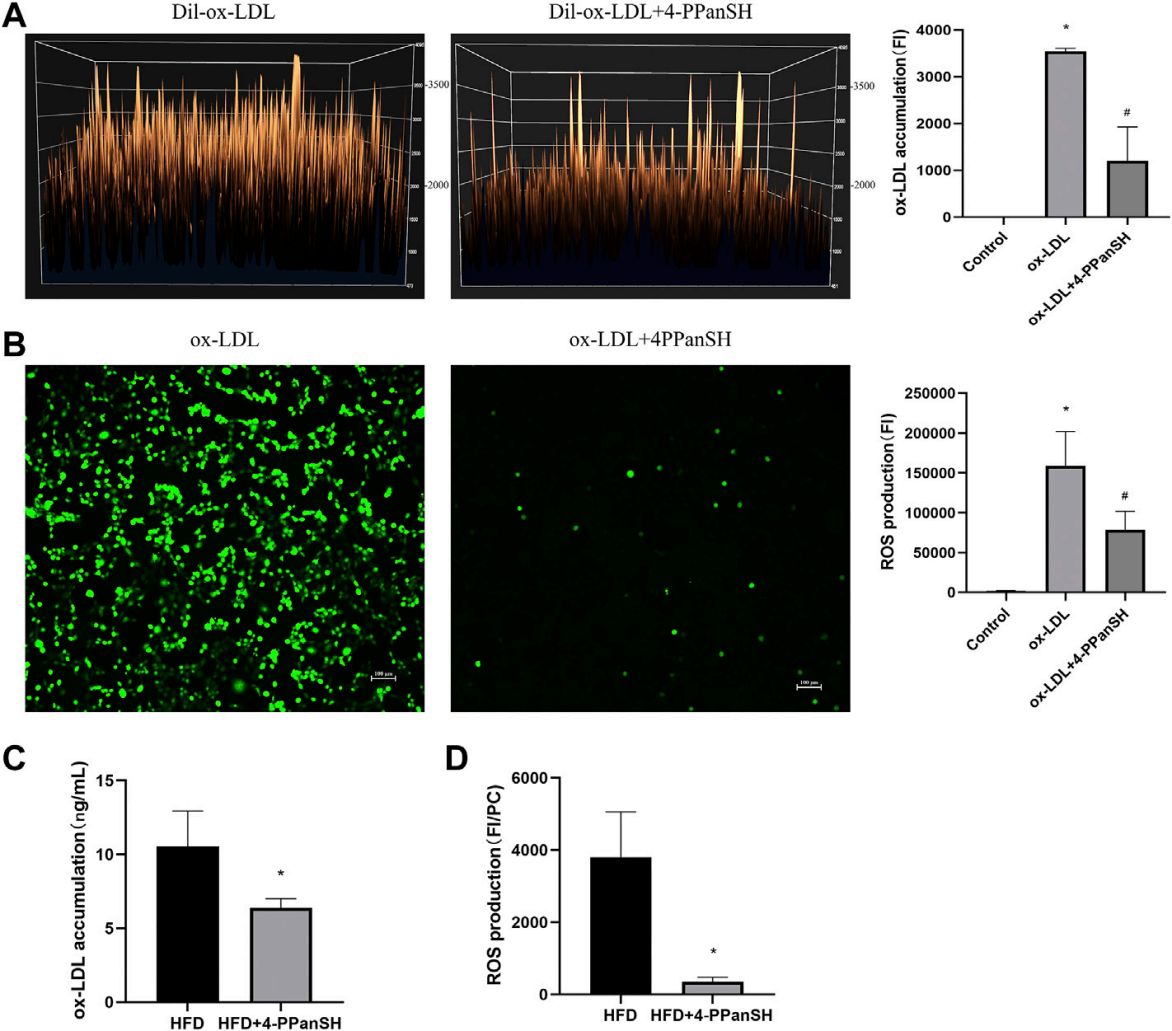
4-PPanSH could inhibit ROS generation. **(A)** Surface fluorescence intensity of endothelial cells after 24 h of treatment with the addition of Dil-labeled ox-LDL. The accumulation of ox-LDL in HUVECs was detected using a fluorescence microplate reader (*n* = 3). **(B)** ROS production detected using a fluorescence microplate reader and fluorescence microscope in HUVECs (*n* = 3). **(C)** The ox-LDL accumulation of vascular tissues in mice was detected by Elisa. 4-PPanSH inhibited the accumulation of ox-LDL in vascular tissues (*n* = 3). **(D)** ROS production of vascular tissues detected using a fluorescence microplate reader in mice. The results were expressed as the ratio of fluorescence intensity to protein concentration. ROS production was inhibited by 4-PPanSH. *Indicates *p* < 0.05 compared to the control group. #Indicates *p* < 0.05 compared to the ox-LDL group. FI means fluorescence intensity. PC means protein concentration.

We examined the relationship between the anti-AS effect of 4-PPanSH and ROS, as oxidative stress injury induced by ROS is one of the leading causes of vascular endothelial injury. The result showed that 4-PPanSH significantly reduced intracellular ox-LDL accumulation (Figure 6A) and inhibited intracellular ROS production (Figure 6B). We also detected the content of ox-LDL and ROS in vascular tissues in mice, and the results showed that 4-PPanSH inhibited ROS production and ox-LDL accumulation (Figures 6C,D). In conclusion, the effects of 4-PPanSH in attenuating vascular endothelial injury may be related to the inhibition of ROS production and accumulation of ox-LDL.

## Discussion

CHD refers to coronary artery stenosis, accounting for 95%–99% of ischemic diseases, with coronary atherosclerosis as the primary etiologic. Currently, the morbidity and mortality of CHD are progressively increasing (Zhang et al., 2020). The lack of effective early detection biomarkers and treatments results in the high morbidity and mortality of CHD (Arroyo-Quiroz et al., 2020). Existing research agrees that the development and progression of AS is a complex mechanism of metabolic disorders involving multiple risk factors; therefore, analyzing the metabolome in the peripheral blood of CHD patients is crucial (Poznyak et al., 2020).

This study used untargeted metabolomics to detect differential metabolites in peripheral blood of CHD patients with various degrees of coronary stenosis. Preliminary data analysis showed an inverse relationship between 4-PPanSH and the extent of coronary lesions, particularly in those at high risk of CHD with plaques in the coronary artery but not with vascular stenosis. Subsequently, the results were again validated with a targeted metabolome. The results indicated that 4-PPanSH, along with the significant diagnostic potential for the high-risk CHD population, can distinguish CHD patients with different degrees of coronary stenosis, and the changes in 4-PPanSH illustrated that the level of 4-PPanSH may be related to the degree of progression of CHD.

4-PPanSH is produced as an intermediate in intracellular CoA synthesis and degradation (Czumaj et al., 2020). In this study, it was found that differential metabolites were produced in the CoA biosynthesis pathway. CoA is an essential metabolic cofactor that is mainly synthesized from pantothenate. The unique structure of CoA allows it to act as a significant acyl carrier and carbonyl activator, producing several different metabolically active thioester derivatives, including acetyl-CoA, malonyl-CoA, and 3-hydroxy-3-methylglutaryl (HMG) CoA. These metabolically active thioester derivatives are involved in the metabolism of lipids and other substances (Czumaj et al., 2020). CoA, an important signaling molecule, and its derivatives have been shown to antagonize platelet function, inhibit excessive platelet aggregation, and regulate the proliferation and vasoconstriction of vascular smooth muscle cells. Therefore, CoA plays an important role in cardiovascular diseases such as AS and hypertension (Davaapil et al., 2014). However, studies have suggested that these CoA effects may be caused by 4-PPanSH (Srinivasan et al., 2015).

Firstly, CoA and its derivatives are charged macromolecules that cannot penetrate the plasma membrane, but 4-PPanSH can freely cross the cell membrane and be used to resynthesize CoA (Naquet et al., 2020). Second, the 4-PPanSH concentration in fresh serum was low, but when CoA was injected into the peripheral blood of mice, the 4-PPanSH concentration increased significantly (Srinivasan et al., 2015). Furthermore, there is evidence that large amounts of CoA can be released into the extracellular space after injury to the cell membrane or cell death in certain conditions such as heart attack, hypertension, and AS, modulating the function of cell surface receptors and acting as a competitive inhibitor of platelet aggregation, possibly preventing excessive thrombosis (Davaapil et al., 2014). In this study, CoA was not detected by either method in the peripheral blood of CHD patients; however, 4-PPanSH had a significantly higher concentration. In addition, it was found that the CoA content was significantly decreased in carotid atherosclerotic plaques compared to the surrounding normal vascular tissue in mice or humans. This may be related to the decomposition of CoA into 4-PPanSH after entering peripheral blood.

As a result, it can be assumed that during AS plaque formation, the vascular endothelium is damaged and releases large amounts of CoA into the peripheral blood, then degrades to 4-PPanSH. The remaining cells then took up 4-PPanSH to resynthesize CoA for a follow-up role or to counteract platelet action as a signaling molecule and further prevent AS plaque formation. 4-PPanSH is significantly depleted as the disease progresses and is not adequately replenished, resulting in a gradual decrease in its concentration. Therefore, we further examined the absorption of 4-PPanSH *in vivo*, and after injecting 4-PPanSH into mice for 24 h, it was significantly decreased in peripheral blood (Supplementary Figure S2), which also proved our conjecture.

It is easy to explain why high levels of 4-PPanSH were found in the peripheral blood of the high-risk (CA) group in this study, but the levels of 4-PPanSH gradually decreased with increasing degrees of coronary artery lesions in CHD patients. Therefore, as a stable form of CoA in peripheral blood, 4-PPanSH is a sensitive indicator of vascular endothelial damage *in vivo*, and 4-PPanSH supplementation could potentially be an effective strategy to prevent and cure AS or CHD. In recent research, 4-PPanSH has been found as a therapeutic candidate for pantothenate kinase-associated neurodegeneration. However, its role in cardiovascular disease remains unclear.

Subsequently, in a mouse model of AS, we experimentally confirmed that 4-PPanSH supplementation effectively inhibited plaque formation. Currently, lowering LDL-C is the primary approach to managing and preventing atherosclerotic cardiovascular disease. In a dose-dependent manner, lowering LDL-C has been shown to effectively reduce the risk of ASCVD (Gupta et al., 2020). Therefore, we first examine common blood lipid markers such as TC and LDL-C in peripheral blood from AS model mice. These findings revealed that 4-PPanSH did not affect the lipid content of peripheral blood.

In addition to LDL-C, a vascular endothelial injury is a crucial event and a reversible condition in the early stage of AS (Zhu et al., 2019). This study demonstrated that 4-PPanSH could effectively reduce the extent of vascular endothelial injury. Vascular endothelial injury is primarily caused by ox-LDL produced by ROS-mediated oxidation modification of LDL. Ox-LDL induces endothelial cell and macrophage dysfunction, alters the local microenvironment of blood vessels, and promotes unstable plaque formation (Hartley et al., 2019). As a result, inhibition of ROS generation by antioxidants is critical to inhibit oxidative modifications of LDL, attenuate endothelial injury, and counteract the onset and development of AS (Kattoor et al., 2019). In this study, 4-PPanSH was found to inhibit ROS production in vascular endothelial cells and ox-LDL uptake by endothelial cells. Therefore, inhibition of ROS production and reduction of endothelial cell injury can be critical for the anti-AS effect of 4-PPanSH.

Therefore, there may be a homeostatic or self-repair capacity *in vivo* during the initial stages of atherosclerosis, in which CoA is released into the peripheral blood after vascular endothelial injury and subsequently decomposes into 4-PPanSH. This part of 4-PPanSH is taken up by vascular endothelial cells to avoid endothelial damage and further progression of atherosclerosis by inhibiting ROS production and accumulation of ox-LDL. But the amount of 4-PPanSH that can be generated by CoA catabolism is limited, and as atherosclerosis progresses, more 4-PPanSH is consumed, resulting in lower levels of 4-PPanSH in peripheral blood. Insufficient subsequent4-PPanSH supplementation may be a key reason for the continuous aggravation of atherosclerosis.

In general, our study confirms that 4-PPanSH is associated with the degree of progression of CHD. The study also found that 4-PPanSH had an inhibitory effect on the formation of AS plaques. This effect might be related to inhibition of ROS production and reduction of ox-LDL uptake by endothelial cells by 4-PPanSH, reducing vascular endothelial injury. However, we synthesized the 4-PPanSH standard according to a reported method. (Srinivasan et al., 2015). But the synthesis of 4-PPanSH is complicated and expensive, which is disadvantageous for its future clinical application, and a more inexpensive and efficient way to synthesize 4-PPanSH is needed in subsequent studies.

## Data Availability

The original contributions presented in the study are included in the article/Supplementary Material, further inquiries can be directed to the corresponding author.
