# Supplementary material for: 4′-phosphopantetheine acts as a potential antioxidant to limit atherosclerotic plaque formation by inhibiting ROS generation
Source: Front Physiol. 2022 Oct 21;13:989105. doi: 10.3389/fphys.2022.989105 (PMC9634529; doi:10.3389/fphys.2022.989105)

Supplementary Material

# Supplementary Data

Table 1. Baseline characteristics of the participants in the discovery cohort (n = 118).

| Characteristics | NON-CA | CA | Mild | Moderate | Severe |
| --- | --- | --- | --- | --- | --- |
| Age | 60.42 | 61.46 | 64.87 | 59.71 | 64.11 |
| Sex male (%) | 53 | 58 | 59 | 59 | 69 |
| HDL-C (mmol/L) | 1.02 | 1.04 | 1.02 | 0.98 | 0.97 |
| LDL-C (mmol/L) | 2.78 | 2.75 | 2.66 | 2.73 | 2.59 |
| TC (mmol/L) | 4.37 | 4.3 | 4.35 | 4.34 | 4.19 |
| TG (mmol/L) | 1.65 | 1.97 | 2.11 | 2.03 | 1.63 |
| Glu (mmol/L) | 7.47 | 7.32 | 6.79 | 7.97 | 7.81 |
| Diabetes history(%) | 66.67 | 64.71 | 51.61 | 50.00 | 57.70 |
| Gensini score | 0 | 0 | 6.47 | 26.39 | 69.07 |

NON-CA (n = 17): normal coronary artery group; CA (n = 12): coronary arteries having plaque but not forming stenosis group; Mild (n = 31): mild stenosis group; Moderate (n = 32): moderate stenosis group; Severe (n = 26): severe stenosis group.

Table 2. Gensini scoring sheet

| Degree of stenosis (%) | Score | Segment | Score |
| --- | --- | --- | --- |
| 1–25 | 1 | LM | 5 |
| 26–50 | 2 | LAD or LCX proximal | 2.5 |
| 51–75 | 4 | LAD mid | 1.5 |
| 76–90 | 8 | LAD apical | 1 |
| 91–99 | 16 | LCX mid and distal | 1 |
| 100 | 32 | RCA | 1 |
|  |  | Branches | 0.5 |

The point for each lesion was the stenosis score multiplied by the lesion site score, and the point for each patient was the sum of all lesion points; Normal coronary arteries (NON-CA) and coronary arteries with plaque but not yet causing vessel stenosis (CA) were counted on a scale of 0; 1–20 as mild lesions (Mild); 21–40 as moderate lesions (Moderate), and greater than 40 as severe lesions (Severe); LM: left main coronary artery; LCX: left circumflex branch; LAD: left anterior descending branch; RCA: right coronary artery.

Figure 1. The structure of the compounds: 4-PPanSH (A)and Carbamazepine-d10 (B).

1. **B.**

**



**

Table 3. Retention time and mass spectrometric parameters about the 4′-Phosphopantetheine and Carbamazepine-d10 in LC-MS/MS method.

| Compounds | Formula | Precursor ion→  Product ion (m/z) | Collision  energy(eV) | Retention  Time (min) |
| --- | --- | --- | --- | --- |
| 4′-Phosphopantetheine  Carbamazepine-d10 | C11H23N2O7PS  C15H2D10N2O | 359.1→216.2  247.1→204.2 | 20  30 | 3.47  4.61 |

Table 4. Baseline characteristics of the participants in the validation cohort (n = 151).

| Characteristics | NON-CA | CA | Mild | Moderate | Severe |
| --- | --- | --- | --- | --- | --- |
| Age | 61.38 | 63.63 | 62.29 | 59.10 | 62.64 |
| Sex male (%) | 48 | 58 | 55 | 62 | 64 |
| HDL-C (mmol/L) | 1.00 | 1.03 | 1.06 | 1.03 | 1.04 |
| LDL-C (mmol/L) | 2.26 | 2.75 | 2.53 | 2.76 | 2.62 |
| TC (mmol/L) | 3.77 | 4.38 | 4.14 | 4.40 | 4.25 |
| TG (mmol/L) | 1.26 | 1.37 | 1.63 | 1.46 | 1.50 |
| Glu (mmol/L) | 5.10 | 5.58 | 5.88 | 7.03 | 6.55 |
| Diabetes history(%) | 62.53 | 62.77 | 61.34 | 58.29 | 59.74 |
| Gensini score | 0 | 0 | 9.82 | 29.95 | 77.03 |

NON-CA (n = 21): normal coronary artery group; CA (n = 24): coronary arteries having plaque but not forming stenosis group; Mild (n = 49): mild stenosis group; Moderate (n = 21): moderate stenosis group; Severe (n = 36): severe stenosis group. In the validation cohort, sample basic information did not show an obvious difference between groups.

Figure 2. The absorption efficiency of 4-PPanSH in mice. A total of 100 ng 4-PPanSH was injected into the mice via the tail vein, and the contents of 4-PPanSH in the mice were detected after 6 h, 12 h, 24 h and 48 h, respectively (n=3). The results showed that 4-PPanSH in mice was significantly decreased after 24 h. *Indicates p <0.05 compared with the 0H group.


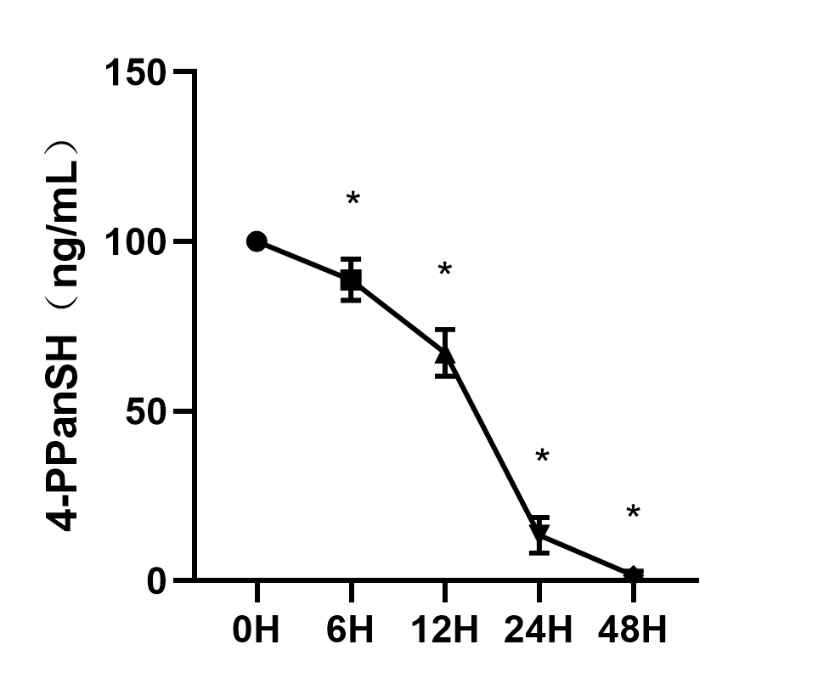

Supplement: Supplementary file 1 [file Table1.DOCX]
